# Engaging Older Minority Adults in a Smartphone-Based Ecological Momentary Assessment Study of Sedentary Behavior

**DOI:** 10.1093/geroni/igaa057.626

**Published:** 2020-12-16

**Authors:** Laurie Kennedy-Malone, Derek Hevel, Kourtney Sappenfield, Heidi Scheer, Christine Zecca, Jaclyn Maher

**Affiliations:** 1 UNCG School of Nursing, Greensboro, North Carolina, United States; 2 University of North Carolina at Greensboro, Greensboro, North Carolina, United States

## Abstract

Minority older adults engage in excessive levels of sedentary behavior (i.e., sitting). Smartphone-based ecological momentary assessment (EMA) methods can provide novel insights into the modeling and prediction of activity-related behaviors. Yet, minority groups report barriers to participating in mobile health research (e.g., distrust, lack of interest, underrepresentation in research). This abstract reports on strategies used to recruit minority older adults and acceptability of an 8-day smartphone-based sedentary behavior EMA study in this population. Researchers partnered with existing community organizations servicing the target population (i.e., independent living communities, congregate meals sites, and churches) and trusted individuals within these organizations to facilitate introduction of the research team/study. In total, 123 older minority adults were recruited, 102 met inclusion criteria and 91 completed the study. During the study, participants answered 6 electronic EMA questionnaires/day and wore an ActivPAL activity monitor continuously. Participants received one-on-one training on these procedures and received check-in calls to monitor progress. Open-ended questions administered at the end of the study revealed the most enjoyable aspects of the study were the ability to learn more about themselves, contributing to science and/or their community, engaging in a new activity followed by receiving financial compensation and having someone checking on their progress. Least enjoyable aspects of the study included frequency of EMA questionnaires, apprehension of missing EMA questionnaires, carrying the smartphone, and difficulty wearing the activity monitor. Almost all participants (95%) expressed interest being contacted for future studies. Implications for future technology-based research regarding minority older adults’ activity-related behaviors will be discussed.

